# Higher protein intake may benefit in patients with prolonged mechanical ventilation

**DOI:** 10.3389/fnut.2024.1449240

**Published:** 2024-10-21

**Authors:** Chiung-Hsin Chang, Chun-Yu Lin, Yu-Lun Lo, Ting-Yu Lin, Chen-Yiu Hung, Meng-Heng Hsieh, Yueh-Fu Fang, Hung-Yu Huang, Shu-Min Lin, Horng-Chyuan Lin

**Affiliations:** ^1^Department of Thoracic Medicine, Chang Gung Memorial Hospital at Linkou, Taoyuan, Taiwan; ^2^College of Medicine, Chang Gung University, Taoyuan, Taiwan; ^3^Department of Thoracic Medicine, New Taipei City Municipal TuCheng Hospital, Chang Gung Medical Foundation, New Taipei City, Taiwan; ^4^Department of Respiratory Therapy, Chang Gung Memorial Hospital at Linkou, Taoyuan, Taiwan

**Keywords:** protein intake, weaning parameter, prolonged mechanical ventilation (PMV), nutrition, PF ratio

## Abstract

**Background:**

Patients with prolonged mechanical ventilation (PMV) is usually associated with muscle wasting and diaphragm weakness, resulting in high medical costs and mortality. Adequate energy and protein intake were beneficial in sarcopenia patients. We aimed to investigate the impact of protein intake in weaning parameters in patients with PMV.

**Materials and methods:**

We enrolled patients with PMV (mechanical ventilation ≥6 h/day for ≥21 days) from a respiratory care center (RCC) of a tertiary medical center from December 2020 to October 2022, and classified them into weaning success and weaning failure groups. The patients’ characteristics, nutrition records, weaning parameters and outcomes were analyzed.

**Results:**

A total of 289 patients were included (mean age 73.5 years). Of the 289 patients, 149 were weaned successfully and 140 were not. The average protein intake was higher in the weaning success group than in the weaning failure group (1.22 ± 0.320 versus 0.99 ± 0.332 g/kg/day, *p* < 0.001). No significant differences were noted in the average calorie intake and whey protein intake between the two groups. RSBI <90 breaths/min/L (OR = 2.38, *p* = 0.045), serum albumin at 4th week ≥3 g/dL (OR = 2.89, *p* = 0.027), daily protein intake ≥1.01 g/kg/day (OR = 8.10, *p* < 0.001), PaO_2_/FiO_2_ (PF) ratio ≥ 300 (OR = 2.56, *p* = 0.027) were independent predictors for weaning from ventilator. Weak positive correlations were found between average protein intake with PF ratio (*r* = 0.1576, *p* = 0.0227) and PaO_2_ (*r* = 0.13359, *p* = 0.0497).

**Conclusion:**

Daily protein intake had positively correlated with PF ratio and had independently benefit for weaning in patients with PMV.

## Background

An estimated 50–74% of all patients in intensive care units (ICUs) receive intubation and mechanical ventilation (1, 2). Of these intubated patients, about 5–10% need prolonged mechanical ventilation (PMV) (2, 3). PMV is defined as ventilation for ≥4 days with tracheostomy placement or ≥21 days without tracheotomy (4). PMV places a heavy burden on healthcare systems and the patient’s family (5, 6), decreases the quality of life, and is associated with poor outcomes in critically ill patients. Furthermore, these patients are associated with a long hospital stay, high 6-month mortality rate, and poor long-term survival; over half of patients with PMV die within 1 year (6, 7).

In patients who survive a critical illness, protein catabolism induced by the illness can lead to decreased respiratory muscle mass, strength and endurance, which may then lead to difficulties in liberation from mechanical ventilation (8). To improve muscle mass and strength, rehabilitation, subjective muscle training and nutrition are equally important. Many studies have shown that adequate protein intake can improve the outcomes of patients with sarcopenia (9). The Liverpool Hope University Sarcopenia Aging Trial (LHU-SAT) also emphasized the importance of concurrent resistance exercise and function exercise to combat age-related muscle weakness (10). Exercise and protein intake have a synergic effect to prevent or improve sarcopenia (11, 12). However, subjective muscle training and effective rehabilitation may not be possible due to weakness or poor consciousness in patients with PMV. Adequate dietary protein intake may be a lower-cost and convenient way to improve sarcopenia in patients with PMV. Many studies have reported that sufficient protein intake can help to increase the mass and strength of muscles (13, 14). In our previous study, we found an association between higher protein intake and successful weaning in patients with PMV (15). Although the timing and amount of calorie and protein intake have been investigated in patients with critical illness (16), few studies have investigated nutritional support in patients with PMV. Therefore, the aim of this study was to investigate correlations of protein intake with weaning parameters, and whether protein intake can benefit in weaning outcomes in patients with PMV.

## Materials and methods

### Patients and data collection

This retrospective study was a single-center analysis of patients with PMV who were admitted to the respiratory care center (RCC) of a tertiary referral teaching hospital in Taiwan between December 2020 and October 2022. The RCC is a specialized weaning unit for patients who could not have successful weaning from the ventilator in the ICU for more than 21 days under a relatively stable clinical condition. The demographic characteristics, underlying diseases, reasons for ICU admission, vital signs and hemodynamic variables, lung mechanics [i.e., tidal volume (TV) and respiratory rate (RR)], hemogram, biochemical variables, arterial blood gas analysis, fraction of inspiration O_2_ (FiO_2_), and nutritional condition (calories, amount and type of protein) were collected from the medical records of the enrolled patients. This study was approved by the Institutional Review Board of Chang Gung Medical Foundation (IRB No. 202200818B0) on June 7th, 2022. All personally identifiable information was encrypted, and the need for patient consent was waived for this study.

The inclusion criteria were: (1) age ≥ 18 years old, and (2) receiving PMV (≥6 h/day for ≥21 days) with an endotracheal tube or tracheostomy. The exclusion criteria were: (1) Acute Physiology and Chronic Health Evaluation II (APACHE II) score ≤10 points, (2) patients who died within 7 days of being admitted to the RCC, (3) patients who were transferred back to the ICU within 7 days of being admitted to the RCC, and (4) total RCC stay <4 weeks for incomplete data analysis.

### Nutrition

Nutritional data including daily protein intake, daily caloric intake, and the feeding regimen for each patient were collected during the RCC stay. All of the patients received bolus enteral feeding with commercial feed via a nasogastric tube. Nutritionist would review and modified the feeding formula at initial RCC admission. Total daily energy and protein requirements were estimated by a dietitian using weight-based formulae (25–30 kcal/kg/day and 0.6–1.5 g/kg/day of protein, respectively) according to the European Society for Clinical Nutrition and Metabolism (ESPEN) and the American Society for Parenteral and Enteral Nutrition (ASPEN) guidelines (17–19). Estimated requirements and feeding regimens were reviewed three times per week by nutritionist according to changes in the patient’s clinical condition. We collected the actual feeding amount according to medical records weekly and analyzed the daily nutrition intake accordingly. Daily whey protein intake was calculated according to the feeding regimen. Enteral feeding was suspended if there were contraindications such as intestinal obstruction, active gastrointestinal bleeding, or severe hemodynamic instability.

### Covariates and outcomes

The following demographic and clinical characteristics were collected: age, sex, height, weight, body mass index, APACHE II score, Sequential Organ Failure Assessment (SOFA) score, hospital length of stay, ICU length of stay, Glasgow Coma Scale (GCS), the presence of tracheostomy, duration of mechanical ventilation, comorbidities, mortality, and weaning parameters. The initial variables were collected at the time of admission to the RCC, and the final variables were collected at week 4 of admission to the RCC or at the time before extubation. The weaning parameters included RR, TV, maximal inspiratory pressure (P_I_Max), rapid shallow breathing index (RSBI), partial pressure of oxygen in the arterial blood (PaO_2_), partial pressure of carbon dioxide in the arterial blood (PaCO_2_), FiO_2_, PF ratio (the ratio of PaO_2_/FiO_2_) and pH. Of these, RSBI was calculated as RR (breaths/min) divided by TV (liters). RR, TV and P_I_Max were determined on a breath-by-breath basis and averaged for the first and last 5 min of the spontaneous breathing trial recording. Arterial blood gas and FiO_2_ were obtained under the pressure support mode of the mechanical ventilator in low setting (FiO_2_: 35%). Nutritional data were collected weekly for 4 weeks, including body weight, daily actual calorie intake, daily actual protein intake, and the type of protein in the feeding regimen (soy, casein, or whey).

The primary outcome of this study was to evaluate the implications of daily protein intake and nutritional status for weaning and their associations with weaning parameters. The secondary outcomes were in-hospital mortality and changes in serum albumin and creatinine levels. Successful weaning was defined as complete liberation from mechanical ventilation (or a requirement for only nocturnal non-invasive ventilation) for 5 consecutive days. Successful weaning and in-hospital mortality were ascertained as of December 2022.

### Statistical analysis

Categorical variables were expressed as count and percentage (%) and compared using the chi-squared test or Fisher’ exact test, as appropriate. Quantitative variables were expressed as mean ± standard deviation (SD) and compared using the Student’s *t-*test or Wilcoxon test, as appropriate. The odds ratio (OR) of weaning success [plus 95% confidence interval (CI)] were estimated using logistic regression analysis adjusted for all covariates. Variables included in multivariate analysis were those that were significant at a two-sided *p-*value of <0.05 in univariate analysis using the stepwise method.

Receiver operating characteristic (ROC) curves were used to identify the optimal cut-off values providing the highest sensitivity and specificity. Correlations of the significant variables were analyzed using Pearson’s correlation coefficients. Statistical analyses were performed using GraphPad Prism version 8 (GraphPad Software, La Jolla, CA, United States) and SPSS version 26 (IBM Inc., Armonk, NY, United States).

## Results

**Figure 1:**
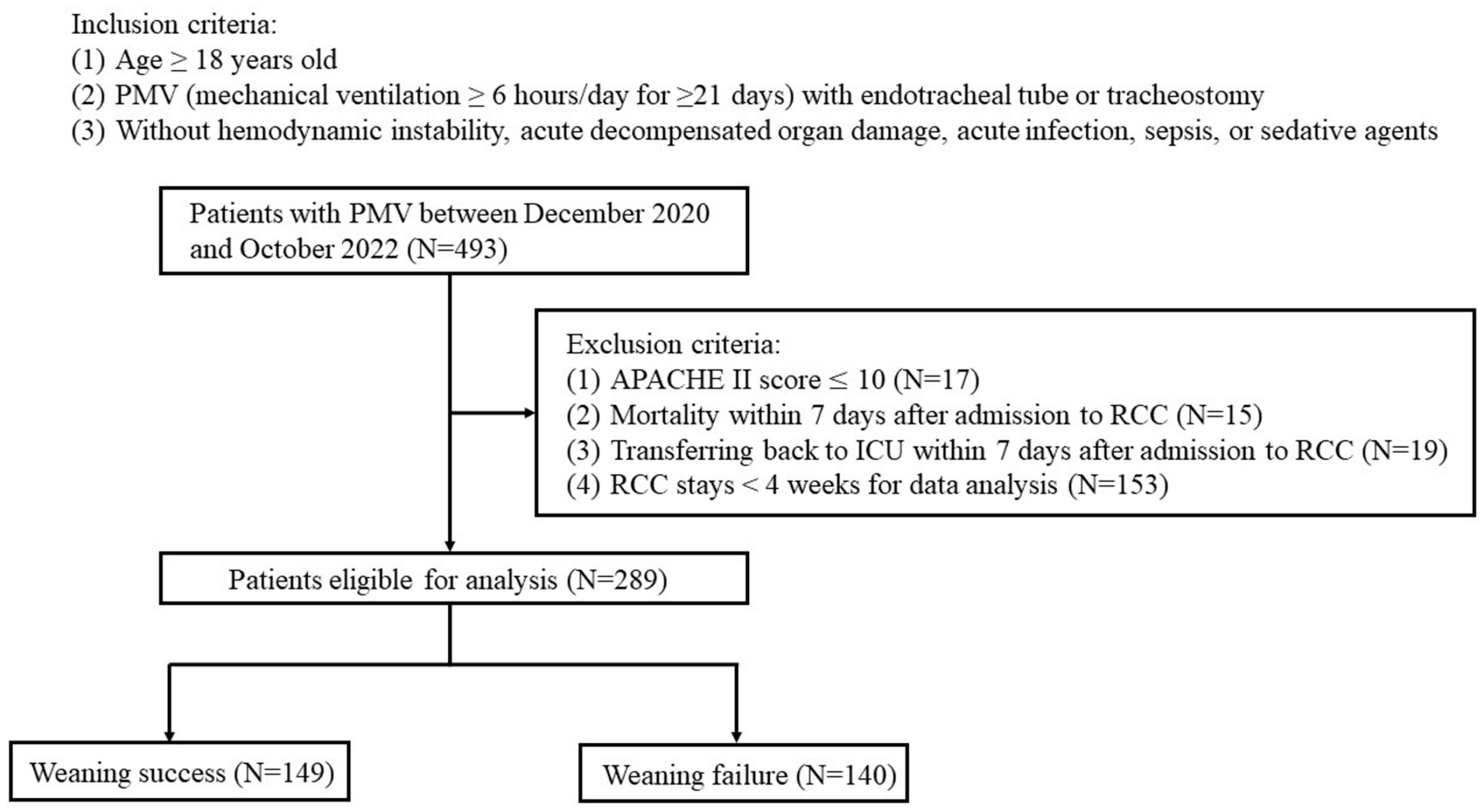
Flow chart of the study design.

A total of 493 patients with PMV were enrolled, of whom 204 were excluded (Figure 1). Of the remaining 289 patients, 149 were successfully weaned (weaning success group) and 140 were not (weaning failure group). The characteristics of the two groups are summarized in Table 1. The mean age of the enrolled patients was 73.5 ± 12.90 years. The mean ICU length of stay before RCC admission was 30.6 ± 19.98 days, the mean hospital stay was 91.9 ± 50.00 days, and the mean duration of mechanical ventilation was 66.3 ± 37.42 days. There were no significant differences in sex, age, body mass index, ICU length of stay, hospital length of stay, and SOFA score between the weaning success and weaning failure groups. However, the APACHE II score was higher in the weaning failure group than in the weaning success group (19.8 ± 5.83 vs. 18.4 ± 4.97, *p* = 0.024).

**Table 1:** Clinical characteristics of the patients with PMV.

	Weaning success *N* = 149 (51.6%)	Weaning failure *N* = 140 (48.4%)	*p*-value
Male (*N*, %)	74 (49.7%)	74 (52.9%)	0.587
Age (year ± SD)	74.6 ± 12.59	72.4 ± 13.17	0.139
BMI (±SD)	23.6 ± 4.60	23.3 ± 4.53	0.519
Reasons for ICU admission			
Septic shock (*N*, %)	19 (12.8%)	21 (15.0%)	0.580
GI bleeding (*N*, %)	4 (2.7%)	0 (0.0%)	0.051
Pneumonia (*N*, %)	66 (44.3%)	80 (57.1%)	0.029
Cardiac arrest (*N*, %)	14 (9.4%)	17 (12.1%)	0.451
Decompensated heart failure (*N*, %)	23 (15.4%)	16 (11.4%)	0.319
Neurologic disorders (*N*, %)	25 (16.8%)	11 (7.9%)	0.022
Cardiac surgery (*N*, %)	6 (4.0%)	7 (5.0%)	0.690
Neurologic surgery (*N*, %)	12 (8.1%)	7 (5.0%)	0.295
Abdominal surgery (*N*, %)	2 (1.3%)	2 (1.4%)	0.950
Comorbidities			
Hypertension (*N*, %)	93 (62.4%)	58 (41.4%)	0.001
Diabetes mellitus (*N*, %)	69 (46.3%)	56 (40.0%)	0.279
Coronary artery diseases (*N*, %)	71 (47.7%)	59 (42.1%)	0.347
Chronic lung diseases (*N*, %)	25 (16.8%)	32 (22.9%)	0.194
Chronic kidney disease (*N*, %)	49 (32.9%)	43 (30.7%)	0.692
Liver cirrhosis (*N*, %)	10 (6.7%)	7 (5.0%)	0.537
Cerebrovascular diseases (*N*, %)	64 (43.0%)	32 (22.9%)	<0.001
Malignancy (*N*, %)	27 (18.1%)	48 (34.3%)	0.002
Hemodialysis (*N*, %)	34 (22.8%)	40 (28.6%)	0.263
APACHE II score	18.4 ± 4.97	19.8 ± 5.83	0.024
SOFA score	7.0 ± 3.02	7.1 ± 3.12	0.860
Duration of mechanical ventilation (days ± SD)	52.7 ± 31.91	80.7 ± 37.56	<0.001
ICU length of stay before RCC admission (day ± SD)	29.5 ± 20.94	31.8 ± 18.92	0.316
Hospital length of stay (day ± SD)	91.6 ± 51.15	92.2 ± 48.91	0.921
Tracheostomy (*N*, %)	60 (40.3%)	99 (70.7%)	<0.001
Mortality (*N*, %)	18 (12.1%)	41 (29.3%)	<0.001
Initial creatinine (mg/dL ± SD)	1.73 ± 1.982	1.65 ± 1.556	0.750
Creatinine at 4th week (mg/dL ± SD)	1.03 ± 1.188	1.22 ± 1.149	0.250
Initial creatinine clearance (ml/min. ± SD)	90.1 ± 69.23	97.8 ± 100.99	0.481
Creatinine clearance at 4th week (ml/min. ± SD)	98. 7 ± 76.77	91.1 ± 87.05	0.504
Initial albumin (g/dL ± SD)	3.00 ± 0.360	2.82 ± 0.423	<0.001
Albumin at week 4 (g/dL ± SD)	3.15 ± 0.383	2.84 ± 0.477	<0.001
Δ albumin (g/dL ± SD)^1^	0.19 ± 0.418	0.02 ± 0.477	0.004
Initial GCS (±SD)^2^	9.3 ± 2.27	9.2 ± 2.60	0.710
Final GCS (±SD)^2^	11.1 ± 2.68	8.2 ± 3.27	<0.001
Initial weaning parameters			
Respiratory rate (breaths/min ± SD)	24.6 ± 7.09	22.9 ± 6.46	0.042
Tidal volume (ml ± SD)	325.1 ± 128.81	304.8 ± 136.90	0.221
P_I_Max (cmH_2_O ± SD)	−28.5 ± 17.90	−27.0 ± 17.60	0.504
RSBI (breaths/min/L ± SD)	89.5 ± 48.31	96.5 ± 66.42	0.331
Final weaning parameters			
Respiratory rate (breaths/min ± SD)	24.1 ± 5.50	24.9 ± 8.30	0.405
Tidal volume (ml ± SD)	337.5 ± 116.28	311.9 ± 143.17	0.151
P_I_Max (cmH_2_O ± SD)	−34.8 ± 12.27	−32.0 ± 12.56	0.098
RSBI (breaths/min/L ± SD)	80.2 ± 37.62	102.4 ± 73.62	0.004
PF ratio (±SD)	337.6 ± 111.40	270.4 ± 118.63	<0.001
PaO_2_ (mmHg ± SD)	121.5 ± 39.81	102.0 ± 34.30	<0.001
PaCO_2_ (mmHg ± SD)	43.1 ± 8.15	45.3 ± 9.67	0.074
pH (±SD)	7.41 ± 0.240	7.41 ± 0.060	0.965
Nutrition parameters			
Average calorie intake (kcal/kg/day ± SD)	26.4 ± 6.82	22.5 ± 7.52	<0.001
Average protein intake (g/kg/day ± SD)	1.22 ± 0.320	0.99 ± 0.332	<0.001
Average whey protein intake (g/kg/day ± SD)	0.56 ± 0.535	0.47 ± 0.443	0.137

^1Δ albumin is the level of albumin at week 4 – initial albumin when the patient was admitted to the RCC. 2For patients with intubation or tracheostomy, the “V” score of Glasgow coma scale were calculated as 1. BMI, body mass index; ICU, intensive care unit; APACHE II score, Acute Physiology and Chronic Health Evaluation II score; SOFA score, Sequential Organ Failure Assessment score; GCS, Glasgow coma scale; SD, standard deviation; RSBI, rapid shallow breathing index; PIMax, maximal inspiratory pressure; PF ratio, PaO2/FiO2 ratio; PaCO2, partial pressure of carbon dioxide in the arterial blood; PaO2, partial pressure of oxygen in the arterial blood; pH, potential of hydrogen.^

Among the reasons for ICU admission, more patients had pneumonia (57.1% vs. 44.3%, *p* = 0.029) and fewer patients had neurologic disorders (7.9% vs. 16.8%, *p* = 0.022) in the weaning failure group than in the weaning success group. There were no significant differences between the two groups in comorbidities except for hypertension, cerebrovascular disease, and malignancy. More patients underwent tracheostomy (70.7% vs. 40.3%, *p* < 0.001) and the mortality rate was higher (29.3% vs. 12.1%, *p* < 0.001) in the weaning failure group than in the weaning success group.

There were no significant differences in creatinine level and creatinine clearance between the two groups either at admission to the RCC or at 4 weeks of admission. The mean serum albumin level at admission to the RCC (3.00 ± 0.360 vs. 2.82 ± 0.423 g/dL, *p* < 0.001), at week 4 (3.15 ± 0.383 vs. 2.84 ± 0.477 g/dL, *p* < 0.001), and the difference between the two values (0.19 ± 0.418 vs. 0.02 ± 0.477 g/dL, *p* = 0.004) were all higher in the weaning success group than in the weaning failure group. Although the initial GCS score was similar between the two groups, the patients in the weaning success group had a higher final GCS score than the weaning failure group (11.1 ± 2.68 vs. 8.2 ± 3.27, *p* < 0.001).

The weaning parameters were similar initially in the two groups. However, at week 4 of RCC admission, the RSBI was significantly lower (80.2 ± 37.62 vs. 102.4 ± 73.62 breaths/min/L, *p* = 0.004) and the PF ratio and PaO_2_ were significantly higher (337.6 ± 111.40 vs. 270.4 ± 118.63, *p* < 0.001; 121.5 ± 39.81 vs. 102.0 ± 34.30 mmHg, *p* < 0.001) in the weaning success group than in the weaning failure group. The average calorie intake (26.42 ± 6.819 vs. 22.54 ± 7.521 kcal/kg/day, *p* < 0.001) and average protein intake (1.22 ± 0.320 vs. 0.99 ± 0.332 g/kg/day, *p* < 0.001) were also significantly higher in the weaning success group than in the weaning failure group. There was no significant difference in average whey protein intake between the two groups.

We determined the cutoff values of the quantitative variables using ROC curve analyses, which showed a final GCS score of ≥10, final RSBI of <90 breaths/min/L, serum albumin level at week 4 of ≥3.00 g/dL, APACHE II score of ≤19, average calorie intake of ≥24.5 kcal/kg/day, average protein intake of ≥1.01 g/kg/day, and PF ratio ≥ 300 were associated with successful weaning (Table 2). Final RSBI of <90 breaths/min/L, serum albumin level at week 4 of ≥3.00 g/dL, average protein intake of ≥1.01 g/kg/day, and PF ratio ≥ 300 were independently predictors for successful weaning.

**Table 2:** Predicting factors associated with successful weaning in patients with PMV.

	Univariate				Multivariate			
	OR	95% CI		*p-*value	OR	95% CI		*p-*value
Final GCS ≥ 10	2.88	1.75	4.75	<0.001	1.67	0.67	4.12	0.270
Final RSBI <90 breaths/min/L	2.00	1.29	2.88	0.015	2.38	1.18	2.98	0.045
Albumin at week 4 ≥3.00 g/dL	3.00	1.77	5.10	<0.001	2.89	1.13	7.38	0.027
APACHE II score ≤19	1.66	1.04	2.66	0.035	1.86	0.82	4.24	0.139
Average calorie intake ≥24.5 kcal/kg/day	2.51	1.56	4.03	<0.001	1.00	0.35	2.89	1.000
Average protein intake ≥1.01 g/kg/day	4.52	2.70	7.58	<0.001	8.10	2.78	23.61	<0.001
PF ratio ≥ 300	2.33	1.31	4.13	0.004	2.56	1.11	5.88	0.027

APACHE II score, Acute Physiology and Chronic Health Evaluation II score; GCS, Glasgow coma scale; RSBI, rapid shallow breathing index; OR, odds ratio; CI, confidence interval.

**Figure 2:**
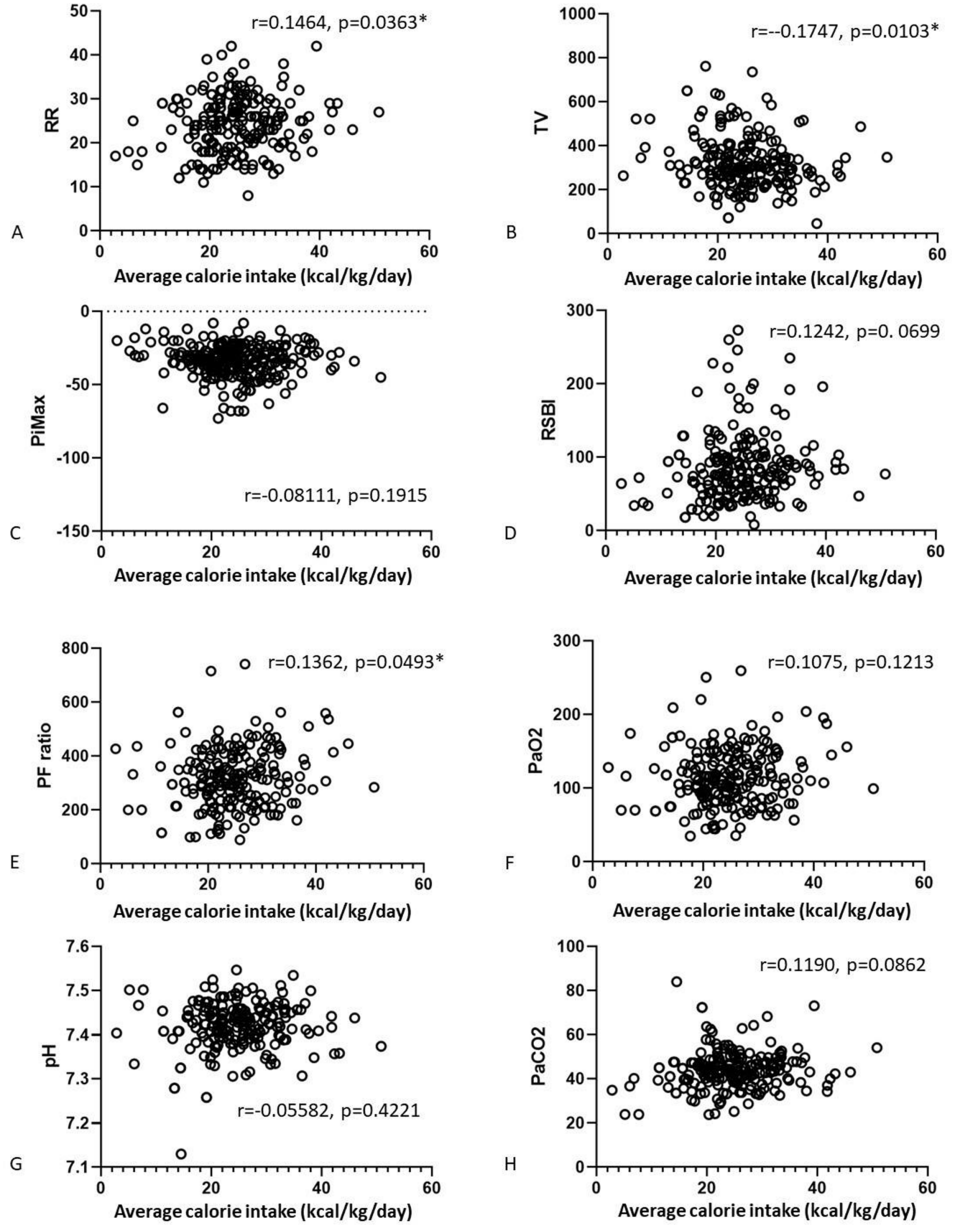
Correlations of the average calorie **(A–D)** and average protein **(E–H)** intake associated with the final weaning parameters.

**Figure 3:**
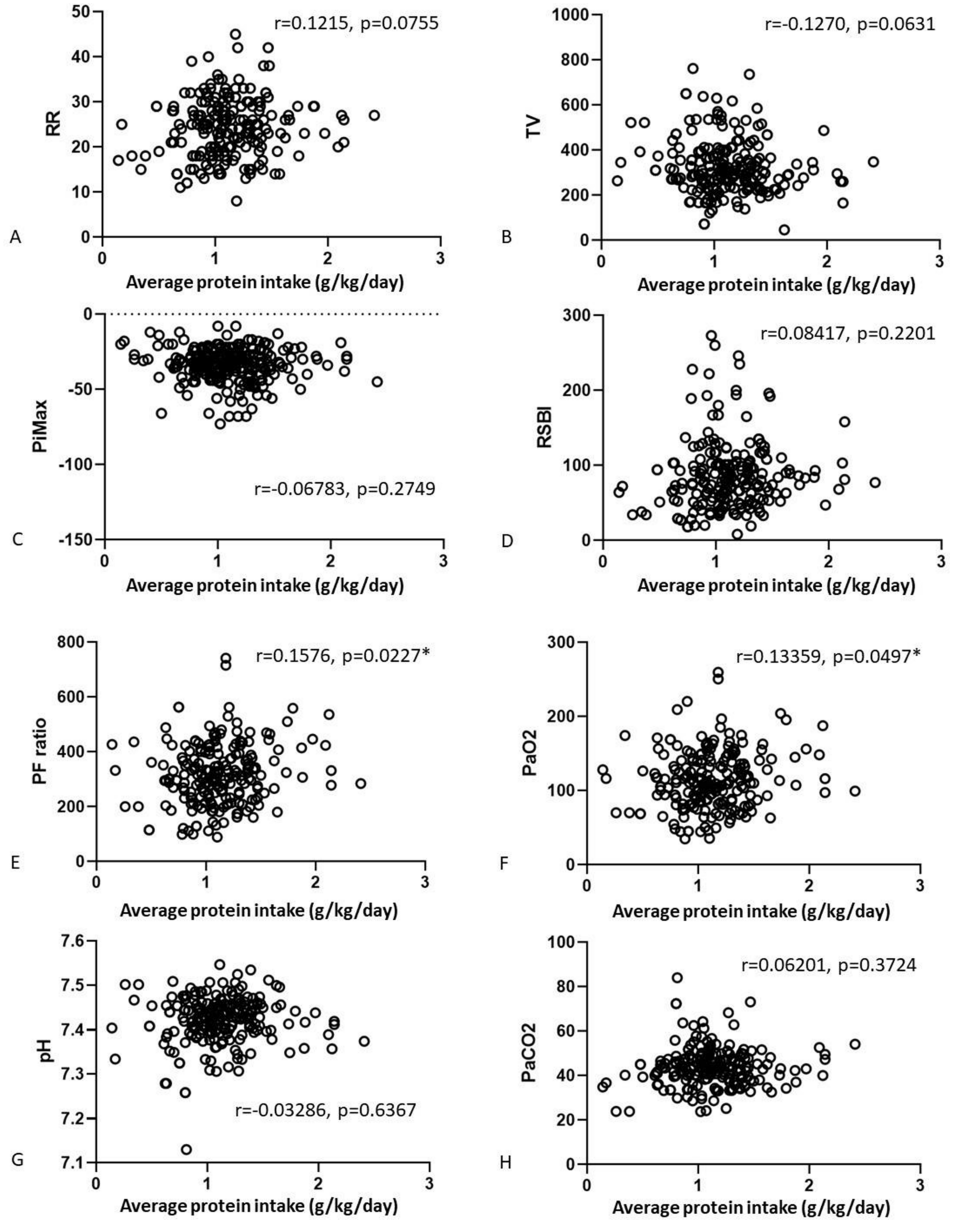
Correlations of the average calorie **(A–D)** and average protein **(E–H)** intake associated with final arterial blood gas parameters.

We also analyzed correlations of the nutrition parameters with the final weaning parameters (Figures 2, 3). There were no correlations of average calorie intake with the PiMax, RSBI, PaO_2_, PaCO_2_. There were positive correlations between average calorie intake with RR (*r* = 0.1464, *p* = 0.0363) and PF ratio (*r* = 0.1362, *p* = 0.0493). Negative correlation between average calorie intake with TV was shown (*r* = −0.17, *p* = 0.0103). There were no significant correlations between average protein intake and RR, TV, PiMax, RSBI, and PaCO_2_. There were positive correlations between average protein intake with PF ratio (*r* = 0.1576, *p* = 0.0227) and PaO_2_ (*r* = 0.13359, *p* = 0.0497). However, these statistically significant correlation coefficients were interpretated as being weak according to Dancey and Reidy’s definition (20).

## Discussion

To the best of our knowledge, this is probably the first study to investigate the associations between high protein intake and weaning parameters in patients with PMV. Our results showed that higher protein intake was associated with successful liberation from mechanical ventilation in the patients with PMV. Daily protein intake also had positive correlation with PF ratio and PaO_2_, presenting with better oxygenation.

Caloric and protein malnutrition have been associated with an increased duration of ventilator days and longer length of ICU stay in critically ill patients, as well as higher rates of morbidity and mortality (21–24). Many studies have indicated that increasing the dietary protein intake can prevent sarcopenia and improve the outcomes of patients with sarcopenia (25–28). Several studies have suggested the optimal timing and amount of calorie and protein intake for patients with acute critical illness (29, 30), however these values for the survivors of critical illness who need PMV are unclear. Because of chronic inflammation, prolonged bedridden status, use of sedatives and muscle relaxants in critically ill patients with mechanical ventilation, they are prone to develop respiratory muscle and limb muscle weakness, which can subsequently delay weaning (31–34). Moreover, prolonged controlled mechanical ventilation may lead to diaphragm disuse and subsequent oxidative stress with protein catabolism in the diaphragm, contributing to diaphragm contractility, dysfunction and atrophy, and thereby increasing the risk of weaning failure (35–38). Zhang et al. (39) reported that intensive nutritional therapy with a target protein intake of up to 2.0 g/kg/day could improve diaphragm atrophy and muscle mass in critically ill patients receiving PMV, but not respiratory mechanical indices or clinical outcomes. In our previous study of 172 patients with PMV, we found an association of high dietary calorie and protein intake with successful liberation from mechanical ventilator (15). In the present study, we extended these findings and investigated the correlations between average calorie/protein intake and weaning parameters. Daily protein intake ≥1.01 g/kg/day, and higher PF ratio were independently beneficial for weaning success. In addition, we found weak, but positive correlations between daily protein intake with PF ratio and PaO_2_. Askanazi et al. (40) reported that ventilatory drive could be enhanced by increasing the intake of nitrogen via continuous parenteral amino acid infusion, and that PaO_2_, minute ventilation and TV increased while PaCO_2_ and respiratory frequency decreased in the high nitrogen intake group. The authors also reported that the increased ventilatory drive observed may not be therapeutic, but depends on the clinical condition of the patients (40). However, another study showed that higher total protein intake failed to affect pulmonary gas exchange or ventilatory response to CO_2_ in postoperative patients (41). Few studies have investigated the effect of nutrition on pulmonary gas exchange and ventilatory drive. Our report found that daily caloric intake had negatively correlated with TV. This may attribute to higher total nutrition intake may increase the intra-abdominal pressure, subsequently decrease the tidal volume and increase the respiratory rate. The highly heterogenous characteristics of our enrolled patients from surgical and medical ICUs and differences between the operators who measured the weaning parameters may also have contributed to the weak correlations. Nevertheless, we still demonstrated that daily protein intake may have benefit in better oxygenation, presenting as positive correlation with PF ratio and PaO_2_.

Some studies have indicated that high-quality proteins (i.e., protein sources with a higher proportion of essential amino acids) such as milk, whey, casein and soy can support muscle protein synthesis (11). However, another study reported that leucine-enriched whey protein supplementation (3 times/day) did not yield further benefits in previously untrained older participants with resistant and functional exercise (10). In the present study, there was no significant difference in average whey protein intake between the weaning success and weaning failure groups. This may be because differences between different proteins (e.g., whey, soy, or casein) are due to postprandial protein metabolism kinetics (42).

Our findings are compatible with a previous study which reported that APACHE II score could not predict successful weaning from PMV (43). In the previous studies, albumin and prealbumin can be both important in the nutrition status survey in the hemodialysis patients (44). Although there was a higher serum albumin level in the weaning success group than in the weaning failure group in this study, many studies have implied that albumin may not be a reliable predictor of successful weaning from a ventilator. A systematic review of 10 studies found no association between outcomes and serum albumin level measured variously on day 5, 7, 10, 12, 14, 15, or 21 of ICU stay (45), while another study showed he patients with serum albumin concentration < 30 g/L have higher 180-mortality whether the nutritional risk and inflammation status are high or not (46). The metabolism of albumin is multifactorial, and the serum level may not reflect the total level in the body, especially as patients with PMV have multiple comorbidities and chronic inflammation (47–49). The amount of protein included in the daily nutritional plan should therefore differ according to the patients’ comorbidities, including those who have acute or chronic renal disease with or without hemodialysis. The impact of serum albumin level was not conclusive in the current study.

Following the ESPEN guidelines (17) to determine the protein requirements of our patients with PMV (about 1.2 g/kg/day), no significant differences in serum creatinine level and creatinine clearance were found between the weaning success and weaning failure groups. A previous study did not find a significant link between high protein intake and the initiation or progression of renal disease in healthy individuals (50), however another study reported that daily protein intake ≥1.4 g/kg/day was associated with higher mortality in patients with impaired kidney function (estimated glomerular filtration rate < 60 mL/min/1.72 m^2^) (51). Few studies have investigated the association between high protein intake and a decline in renal function in patients with PMV. Our results suggest that it is safe to administer a high protein diet according to the ESPEN guidelines in patients with PMV within 4 weeks of admission to an RCC.

There are several limitations to this study. First, this is a retrospective study. The actual intake may have some bias according to the daily nutritional record. Moreover, the urine urea nitrogen was not calculated routinely in our study, thus we could not evaluate the nitrogen balance in our patients. The results in our study only showed associations but not strong correlation between higher dietary protein intake and successful liberation from mechanical ventilation Second, the patients enrolled in this study were from surgical and medical ICUs, which contributed to the high variability of the patients’ characteristics and the weaning outcomes. This may have resulted in selection biases and diminished generalizability. Finally, the short follow-up period may not reflect the long-term outcomes of patients with PMV. Further prospective case–control studies are warranted to confirm our findings.

## Conclusion

Daily protein intake was independently associated with successful liberation from mechanical ventilation in the patients with PMV, and presenting positive correlation with better oxygenation.

## Data Availability

The raw data supporting the conclusions of this article will be made available by the authors, without undue reservation.
